# Identification of Early Recurrence Factors in Childhood and Adolescent B-Cell Acute Lymphoblastic Leukemia Based on Integrated Bioinformatics Analysis

**DOI:** 10.3389/fonc.2020.565455

**Published:** 2020-09-29

**Authors:** Yan Huang, Jiazheng Li, Yanxin Chen, Peifang Jiang, Lingyan Wang, Jianda Hu

**Affiliations:** Fujian Provincial Key Laboratory of Hematology, Fujian Institute of Hematology, Fujian Medical University Union Hospital, Fuzhou, China

**Keywords:** acute lymphoblastic leukemia, early recurrence, genetic risk score model, differential gene expression, microRNA

## Abstract

Over the past 50 years, great progress has been made in the diagnosis and treatment of acute lymphoblastic leukemia (ALL), especially in pediatric patients. However, early recurrence is still an important threat to the survival of patients. In this study, we used integrated bioinformatics analysis to look for biomarkers of early recurrence of B-cell ALL (B-ALL) in childhood and adolescent patients. Firstly, we obtained gene expression profiles from the Therapeutically Applicable Research to Generate Effective Treatments (TARGET) database and the Gene Expression Omnibus (GEO) database. Then, we identified differentially expressed genes (DEGs) based on whether the disease relapsed early. LASSO and Cox regression analysis were applied to identify a subset of four genes: *HOXA7, S100A11*, S100A10, and *IFI44L.* A genetic risk score model was constructed based on these four optimal prognostic genes. Time-dependent receiver operating characteristic (ROC) curves were used to evaluate the predictive value of this prognostic model (3-, 5-, and 10-year AUC values >0.7). The risk model was significantly associated with overall survival (OS) and event-free survival in B-ALL (all *p* < 0.0001). In addition, a high risk score was an independent poor prognostic risk factor for OS (*p* < 0.001; HR = 3.396; 95% CI: 2.387–4.832). Finally, the genetic risk model was successfully tested in B-ALL using an external validation set. The results suggested that this model could be a novel predictive tool for early recurrence and prognosis of B-ALL.

## Introduction

Acute lymphoblastic leukemia is the most common cancer in children, and includes both B-cell and T-cell lineages. B-ALL accounts for about 85% of pediatric ALL (1). With advances in chemotherapy and hematopoietic stem cell transplantation, the cure rate in childhood ALL is currently around 90% (2). The prognosis for adolescent patients aged 15 to 20 years receiving pediatric protocols is similar to that of children, with a 5-year OS of 87.9% (3). In spite of significant progress in long-term survival, 15–20% of patients will suffer a recurrence, which is an important factor for increases in mortality (4). Relapses occurring less than 18 months after diagnosis are defined as very early relapses, relapses appearing between 18 months of initial diagnosis and 6 months after cessation of frontline therapy is regarded as an early relapse, and relapses developing after 6 months of cessation of frontline treatment are classified as late relapses (5). The time from diagnosis to relapse is an independent risk factor for overall survival (6), and the risk ratios for adverse outcome in patients with late, early, and very early relapse were 1, 2.4, and 2.9, respectively (*p* < 0.001) (5). Patients with late recurrence had better survival rates than those who relapsed earlier (OS: 45–73% vs. 22–38%) (7–9). Thus, identifying patients with a high risk of earlier relapse is crucial to improving prognosis.

Genomic abnormalities have been confirmed to be related to treatment response and disease relapse in acute leukemia (10–12). An understanding of genetic abnormalities can guide the selection of subsequent regimens after the induction of remission. Technological progress has led to more detailed genetic profiling of leukemia, including DNA sequence abnormalities, gene expression abnormalities, chromosomal rearrangements, and abnormal epigenetic modifications. It has been suggested that the development of gene expression profiles for acute leukemia can improve the prediction of prognosis (13). For example, Ng et al., established a 17-gene stemness score for rapid determination of risk in acute myeloid leukemia (AML) (14). Overexpression of Wilms tumor 1 gene indicates an adverse prognosis and poor response to treatment in AML (15). In Ismail et al.’s study, the expression level of the gene *BIRC6* was an adverse risk factor in acute childhood leukemia (16). Therefore, we aimed to investigate the relationship between genetic profiling and early relapse of childhood B-ALL.

In this study, we identified differentially expressed genes (DEGs) between individuals with early recurrence and no early recurrence of B-cell ALL, using data from the GEO and TARGET public databases. We constructed a prognostic risk model based on DEGs, using regression analysis. We verified the prognostic value of this risk model using an external validation set from the TARGET database. Using the potential biomarkers of early relapse in B-cell ALL, we provided a new approach to early diagnosis and treatment of patients at high risk of recurrence.

## Materials and Methods

### Database

We downloaded one dataset (GSE13576) from the GEO database^1^. The data from GSE13576 were based on the GPL570 platforms (Affymetrix Human Genome U133 Plus 2.0 Array) and included data on 197 pediatric ALL patients with mRNA expression information. The mRNA expression dataset of 486 B-cell ALL patients and their corresponding clinical information were obtained from the TARGET database^2^. This dataset included 370 microarray data points and 116 mRNA-seq data points. The data of 370 cases were based on the GPL570 platform. miRNA-seq was performed in 53 of 486 patients from the TARGET dataset.

### Analysis of Differentially Expressed Genes

The definition of early recurrence in this study was less than 24 months from diagnosis. Data from 370 childhood and adolescent B-cell ALL patients from the TARGET database, and 197 pediatric patients from GSE13576 were classified into early relapse and non-early relapse groups. Of the 370 patients from the TARGET database, 127 had early recurrence and 243 did not have early recurrence. In the GSE13576 dataset, 24 patients were in the early relapse group, and 173 patients belonged to the no early recurrence group. We used the limma R package to normalize the data and performed gene differential analysis by comparing the two groups in the R computing environment (Version 3.6.2). The differences in the gene expression levels were adjusted for multiple testing using the Benjamini-Hochberg method. Genes with a *p*-value < 0.05 and | Log2(fold change) | > 0.5 were defined as DEGs in this study. Finally, a volcano plot and heatmap of the DEGs was drawn using the ggrepel and heatmap R packages, respectively. The Venn diagrams of the DEGs were drawn using the website tool Venny 2.1.0^3^.

### Selection of Candidate Genes

The 370 samples from the TARGET database were randomly separated into training and test (6:4) sets for constructing and validating the prognostic models, using the base R package. Univariate Cox regression analyses were used to explore the correlations between the overall survival of B-cell ALL patients and the expression level of DEGs in training set (*n* = 222). The genes with a *p-*value < 0.01 were selected as candidate prognosis-related genes. To further investigate the candidate genes, least absolute shrinkage and selection operator (LASSO) regression analysis was applied using the glmnet R package. This algorithm minimizes the usual sum of squared errors with tenfold cross-validation, and identifies the optimal lambda value. The genes with coefficient are related to prognosis. The optimal prognostic genes derived from the LASSO regression model were validated using the Oncomine website^4^. We selected genes associated with early recurrence as hub genes with which to construct the prognostic model. Unpaired *t*-tests were used to compare the levels of gene expression between groups, using GraphPad Prism (Version 7.03).

### Genetic Risk Score Model Construction

The gene-related prognosis model in this study was established using the training set. We applied a multivariate COX regression model to assess the role of hub genes as independent prognostic risk factors. Then, a four-gene-based genetic risk score model was constructed. The risk score formula is as follows:

$$\text{Risk}\,\text{score}=\sum_{i=1}^{n}\beta \,i\times E\,x\,p\,i$$

The β in the above formula is the regression coefficient of each gene, and the Exp is the expression level of the gene. The cut-off value of the risk score was defined as the median score. The 222 patients were separated into low- and high-risk groups based on the threshold. To evaluate the predictive power of this risk score model, we used the survival ROC R package to plot time-dependent receiver operating characteristic (ROC) curves, and calculated the area under the ROC curves (AUC).

### Validation of the Genetic Risk Score Model

For internal and external validation, the testing set (*n* = 148), total set (*n* = 370) and external validation set (*n* = 116) were used to assess the predictive power of the genetic prognostic model for B-ALL. The risk score of each patient was calculated using the regression coefficients of four genes in the training set. The patients were divided into high- and low-risk groups according to the median risk score of the training set. Time-dependent ROC curves were plotted for the validation sets. Survival analysis of the patients was constructed using the R software, with the survival and survminer packages. We created Kaplan-Meier plots to illuminate the correlations between the genetic risk scores and the survival index of patients, including overall survival and event-free survival (17). Univariate and multivariate COX regression analyses were used to test whether the genetic risk score model is an independent prognostic risk factor relative to the clinical characteristics of the total set. Statistical significance was tested using log-rank tests. The prognostic risk factors were analyzed using Cox Regression with the survival R packages. A *p*-value < 0.05 was considered to be statistically significantly different.

### Analysis of Differentially Expressed MicroRNAs

For studying the regulation of gene expression, we obtained miRNA-seq data for 53 B-ALL patients from the TARGET dataset. Eight patients relapsed within 24 months, and were included in the early recurrence group, and the remaining 45 patients were included in the no early recurrence group. The edgeR R package was used to identify differentially expressed miRNAs (DEMs) between the two groups. Genes with a adjusted p-value < 0.05 and | Log2(fold change) | > 0.5 were defined as DEMs. Volcano plots were performed using R packages.

### mRNA-miRNA Network Construction

The miRNAs which were target DEGs were predicted by the miRWalk^5^ and DIANA-Tarbase v.8 databases (18). We selected the miRNAs that existed in both prediction databases to construct an mRNA-miRNA network using Cytoscape software (Version 3.7.2).

## Results

### Characteristics of B-ALL Patients in the Total Set From the TARGET Database

To determine the association of genetic profiles with early recurrence in B-ALL, we obtained microarray profiles from 370 patients, and their clinical information, from the TARGET database. Among them, 225 (60.8%) patients were male and 145 (39.2%) were female. The age at initial diagnosis ranged from 1 to 20 years, with a median age of 10 years. The clinical characteristics of the patients are listed in Table 1, including cytogenetics, minimal residual leukemia (MRD) at day 29 after induction, and evaluation of the central nervous system (CNS) at diagnosis. The median follow-up time was 8.9 years for all 370 B-ALL patients. At the end of follow-up, 246 patients had relapsed (127 relapsed within 24 months since diagnosis) and 169 patients had died. The median OS and EFS time were 9.6 years (range, 0.5–15.7 years) and 3.3 years (range, 0.1–15.7 years), respectively.

**TABLE 1:** Characteristic of patients.

**Characteristic**	**Number of cases**	**Percentages (%)**
**Patients**	370	100
**Median age, years (range)**	10 (1–20)	/
**Sex**		
Female	145	39.2
Male	225	60.8
**WBC (×10^9^/L)**		
≥50	152	41.1
<50	218	58.9
**Cytogenetics**		
*t*(9;22)/BCR-ABL fusion gene	2	0.5
*t*(4;11)/AF4 fusion gene	14	3.8
Trisomy 4/10/17	38	10.3
Others	228	61.6
Unknown	88	23.8
**Down’s Syndrome**		
Positive	32	8.7
Negative	272	73.5
Unknown	66	17.8
**CNS^#^**		
CNS1	291	78.6
CNS2	57	15.4
CNS3	22	6.0
**MRD (Day 29)**		
≥0.01%	164	44.3
<0.01%	199	53.8
Unknown	7	1.9
**Protocol**		
POG9906	200	54.1
AALL^&^	170	45.9

*#CNS, Central Nervous System, CNS1 = CNS negative, CNS2 ≤ 5 WBC/μL in CSF with blasts, CNS3 = CNS positive; ^&^AALL includes protocol AALL0232, 0331 and 03B1. ‘/’ means the characteristic of median age is not suitable for percentage calculation.*

### Identification of DEGs Based on Disease Recurrence

**FIGURE 1:**
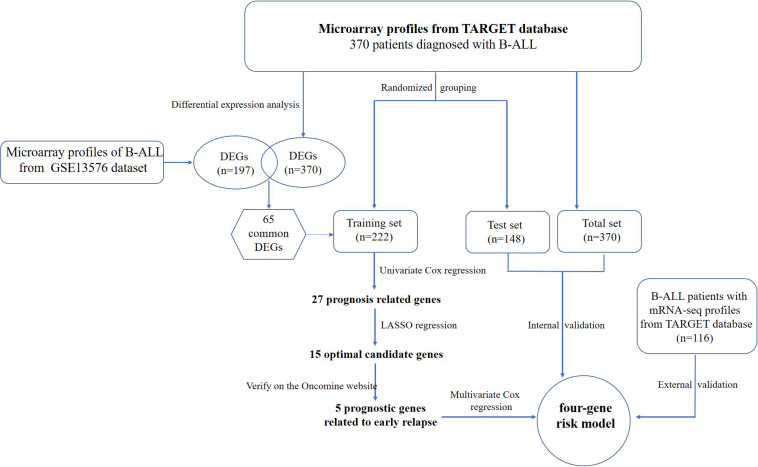
Flowchart of risk model construction.

**FIGURE 2:**
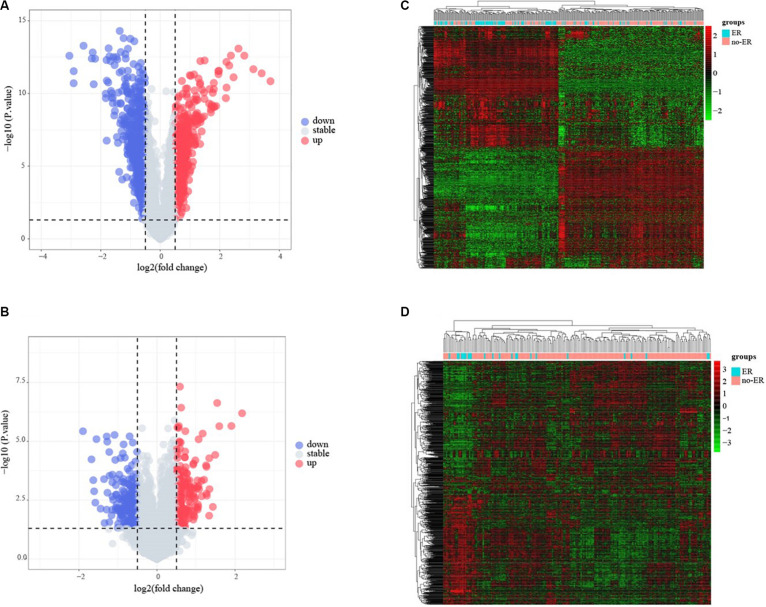
Identification of early recurrence-associated DEGs in pediatric B-cell acute lymphoblastic leukemia. Volcano plot of DEGs from Total Set **(A)** and GSE13576 **(B)**. Genes with *p* < 0.05 and | log2(FC)| > 0.5 are shown in red (upregulated genes) and green (downregulated genes). **(C,D)** Heatmap of DEGs for two datasets, ER means early relapse, no-ER means no early relapse.

**FIGURE 3:**
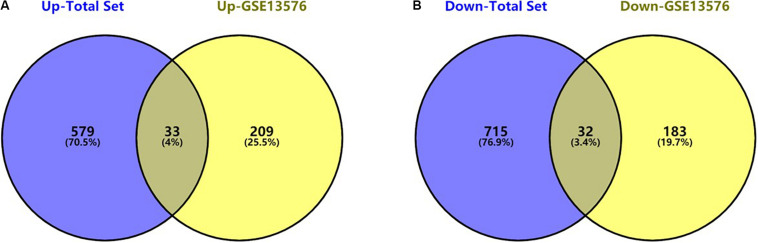
Commonly changed DEGs in Total Set and GSE13576. **(A)** Upregulated genes in two datasets (33 common DEGs) **(B)** Downregulated genes in two datasets (32 common DEGs).

The flowchart of the construction of our risk model is shown in Figure 1. To identify the DEGs, we analyzed the gene expression data of 370 precursor B-cell ALL patients obtained from TARGET, and 197 pediatric ALL patients from GSE13576. Using a threshold of p < 0.05 and | fold change| > 0.5, we identified a total of 1359 DEGs in the TARGET dataset, including 747 upregulated genes and 612 downregulate genes. There were 457 DEGs in GSE13576, including 215 upregulated genes and 242 downregulate genes. The DEGs of the early relapse vs. non-early relapse groups in these two datasets are shown in the volcano plot and heatmap, separately (Figure 2). Through integrated analysis, we identified 33 commonly upregulated genes and 32 downregulated genes from the TARGET and GEO datasets (Figure 3 and Table 2).

**TABLE 2:** The common DEGs identified among GSE13576 and total set.

**Gene Symbol (upregulated DEGs)**
TOMM22, COTL1, S100A11, TSPO, HOXA7, CTSC, BIK, TUBB2A, RAPGEF5, SUCLG2, SNX10, TOP1MT, CRIP1, S100A10, ANXA2P2, CXXC5, S100A4, CAMK2D, IGFBP7, S100A6, DAD1, CPVL, PKIG, TUBB6, HK2, WIPI1, MEIS1, LGALS1, TST, CHN2, MAP7, PPP1R14A, CLEC11A
**Gene Symbol (downregulated DEGs)**
ZNF91, GFOD1, SFMBT2, ZNF704, SIPA1L2, GBP4, CD69, HERC5, IFI44, TCF4, SHANK3, DAPK1, IFI44L, MYO5C, MRC1, ST3GAL6, MAN1A1, NAV1, FHIT, CYP46A1, STK32B, COL5A1, MX1, PDE4B, S100Z, RASD1, POU4F1, ITGA6, MDK, P2RY14, DDIT4L, DNTT

### Construction of the Prognostic Risk Score Model Using the Training Set

**FIGURE 4:**
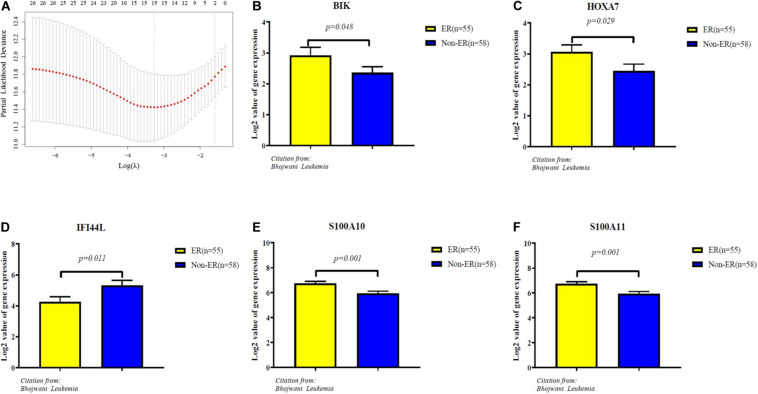
Identification of five significantly prognostic genes related to early relapse. **(A)** LASSO regression with tenfold cross-validation obtained 15 prognostic genes using optimal lambda value. **(B–F)** The expression level of prognostic genes between ER and no-ER groups. ER means early relapse, no-ER means no early relapse.

The 65 common DEGs (including 33 upregulated and 32 downregulated genes) were analyzed for prognostic value using univariate Cox regression on the training set. As shown in Table 3, **27** common DEGs had a p-value < 0.01, including 17 with a Hazard Ratio (HR) > 1 and 10 with HR < 1. Using the LASSO algorithm, the optimal lambda value was 0.038, and 15 of the 27 DEGs were selected as potential prognosis-related genes (Figure 4A and Table 4). Subsequently, we searched for the relationship between 15 common DEGs and early relapse using the Oncomine database. As shown in Figures 4B–F, the gene expression of BIK, HOXA7, S100A10 and S100A11 were significantly higher in B-ALL patients who relapsed within 24 months (both p < 0.05). The gene IFI44L had lower expression levels in early relapse patients (p < 0.05). These five genes associated with early recurrence were selected to build a genetic risk score model for B-ALL patients, using multivariate Cox regression. The risk model was constructed from the four optimal genes: HOXA7, S100A10, S100A11, and IFI44L. The genetic risk score model is as follows:

**TABLE 3:** The result of univariate Cox regression in training set.

**Gene**	**HR**	***z***	***p*-value**
HOXA7	1.538	5.805	6.43E-09
S100A11	1.476	5.324	1.02E-07
TOMM22	1.500	5.195	2.05E-07
SFMBT2	0.825	−5.175	2.27E-07
ZNF704	0.731	−5.113	3.18E-07
COTL1	1.384	5.050	4.42E-07
S100A6	1.498	4.947	7.55E-07
S100A4	1.524	4.885	1.04E-06
TSPO	1.514	4.609	4.05E-06
S100A10	1.350	4.455	8.41E-06
MDK	0.794	−4.058	4.95E-05
ANXA2P2	1.342	3.983	6.8E-05
HERC5	0.639	−3.938	8.21E-05
CYP46A1	0.808	−3.767	0.000165
CRIP1	1.239	3.686	0.000227
CTSC	1.288	3.668	0.000244
BIK	1.202	3.437	0.000588
HK2	1.322	3.369	0.000754
CXXC5	1.226	3.250	0.001155
GFOD1	0.713	−3.029	0.002457
TOP1MT	1.279	2.915	0.003553
CHN2	1.173	2.906	0.003658
IFI44L	0.896	−2.881	0.003967
LGALS1	1.136	2.750	0.005964
GBP4	0.780	−2.688	0.007177
MX1	0.837	−2.648	0.008097
IFI44	0.856	−2.639	0.008306
ZNF91	0.787	−2.410	0.015962
IGFBP7	1.144	2.329	0.019885
DAD1	1.208	2.260	0.023824
TUBB6	1.124	2.244	0.024804
RAPGEF5	1.303	2.182	0.029118
WIPI1	1.168	2.104	0.035405
PPP1R14A	1.103	2.032	0.042198
SIPA1L2	0.918	−1.968	0.049121
MEIS1	1.100	1.952	0.050895
TUBB2A	1.128	1.951	0.051024
DAPK1	0.891	−1.945	0.051819
CPVL	1.100	1.890	0.058766
CAMK2D	1.118	1.776	0.075672
COL5A1	0.918	−1.752	0.079833
MAP7	1.111	1.743	0.081344
TST	1.120	1.713	0.08663
DDIT4L	0.926	−1.668	0.09529
FHIT	0.892	−1.665	0.095844
ST3GAL6	0.908	−1.643	0.10029
SUCLG2	1.143	1.625	0.104209
PKIG	1.113	1.591	0.111563
SNX10	1.128	1.506	0.132115
CLEC11A	1.091	1.503	0.132803
MYO5C	0.873	−1.485	0.137488
MRC1	0.937	−1.359	0.174022
P2RY14	0.943	−1.083	0.278976
SHANK3	0.970	−0.942	0.346027
POU4F1	0.957	−0.855	0.39244
S100Z	0.954	−0.833	0.404886
NAV1	0.949	−0.787	0.431054
STK32B	1.039	0.662	0.508211
TCF4	0.964	−0.577	0.564058
PDE4B	0.973	−0.505	0.613274
RASD1	0.972	−0.469	0.638796
ITGA6	1.023	0.438	0.661388
DNTT	0.981	−0.402	0.687464
MAN1A1	0.984	−0.264	0.791807
CD69	1.012	0.155	0.876639

**TABLE 4:** The result of LASSO regression in training set.

**Gene Symbol**	**Coefficients**
ANXA2P2	0
BIK	0.065
CHN2	0.059
COTL1	0
CRIP1	0
CTSC	0
CXXC5	0.016
CYP46A1	0
GBP4	−0.020
GFOD1	0
HERC5	−0.027
HK2	0
HOXA7	0.239
IFI44	−0.055
IFI44L	−0.048
LGALS1	0
MDK	−0.069
MX1	−0.052
S100A10	0.006
S100A11	0.148
S100A4	0
S100A6	0.159
SFMBT2	−0.001
TOMM22	0
TOP1MT	0
TSPO	0
ZNF704	−0.068

$$\begin{array}{c} \text{Risk}\,\text{score}=\left(0.329*\text{expression}\,\text{value}\,\text{of}\,H\,O\,X\,A\,7\right)+\\ \left(0.136*\text{expression}\,\text{value}\,\text{of}\,S\,100\,A\,10\right)+\\ \left(0.275*\text{expression}\,\text{value}\,\text{of}\,S\,100\,A\,11\right)+\\ \left(-0.151*\text{expression}\,\text{value}\,\text{of}\,I\,F\,I\,44\,L\right) \end{array}$$

**FIGURE 5:**
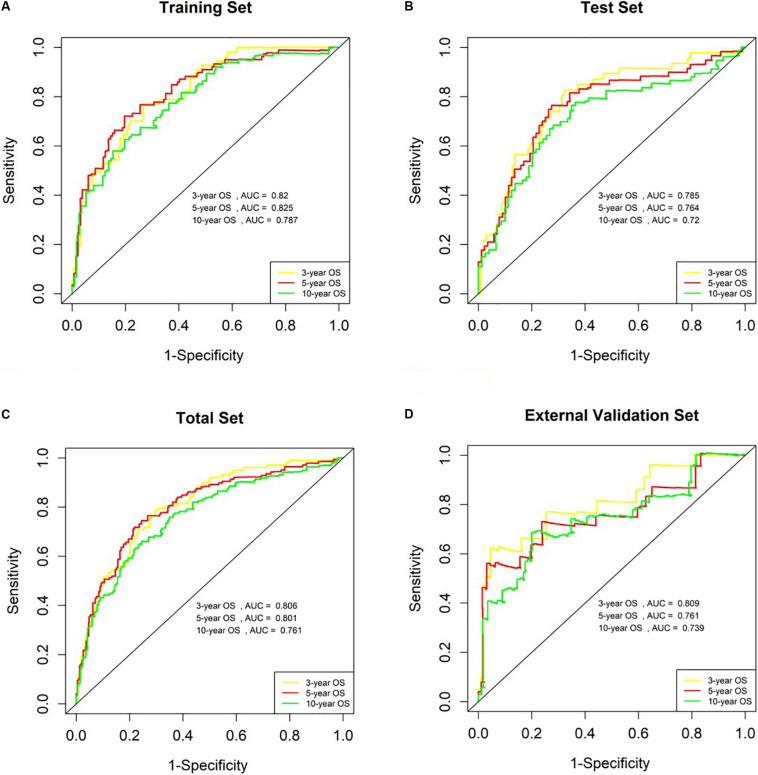
Analysis of genetic risk score model in B-ALL patients. **(A–D)** Time-dependent ROC curves show AUC values at 3-year, 5-year and 10-year OS rate in training set, Test Set, Total Set, and External Validation Set, respectively.

The AUC values for OS at 3 years, 5 years and 10 years were 0.820, 0.825 and 0.787 in the training set, respectively (Figure 5A). The risk score ranged from 1.535 to 6.539, and we selected the median risk score as the cut-off value (4.045). If the risk score was greater than the cut-off value, the patient was defined as belonging to the high-risk group, otherwise the patient was classified in the low-risk group.

### Verification of the Prognostic Risk Score Model in the Validation Set

**FIGURE 6:**
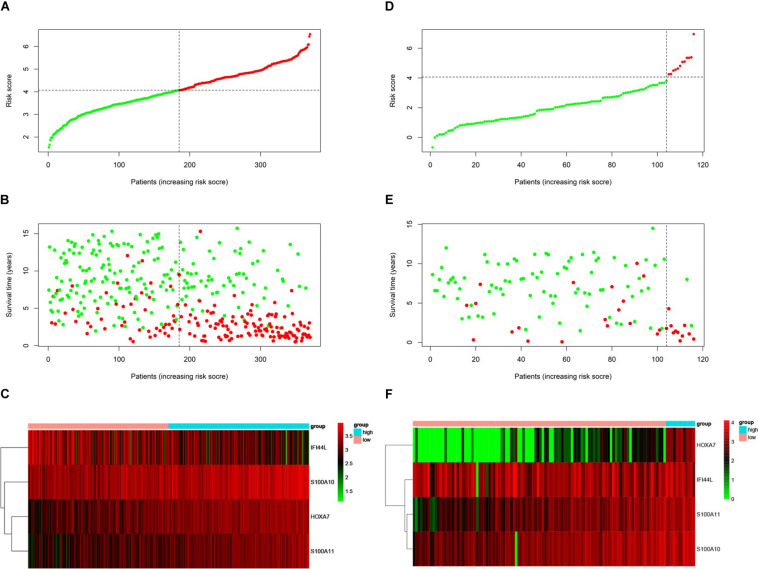
Prognosis of high-risk and low-risk group in B-ALL patients. Risk score distribution of high-risk (red) and low-risk (green) group in B-ALL patients from Total Set **(A)** and External Validation Set **(D)**. Scatter plots show survival status in Total Set **(B)** and External Validation Set **(E)**, red plots represent dead and green plots represent alive. Heatmap of the expression profiles of the four prognostic genes in low- and high-risk group in Total Set **(C)** and External Validation Set **(F)**.

**FIGURE 7:**
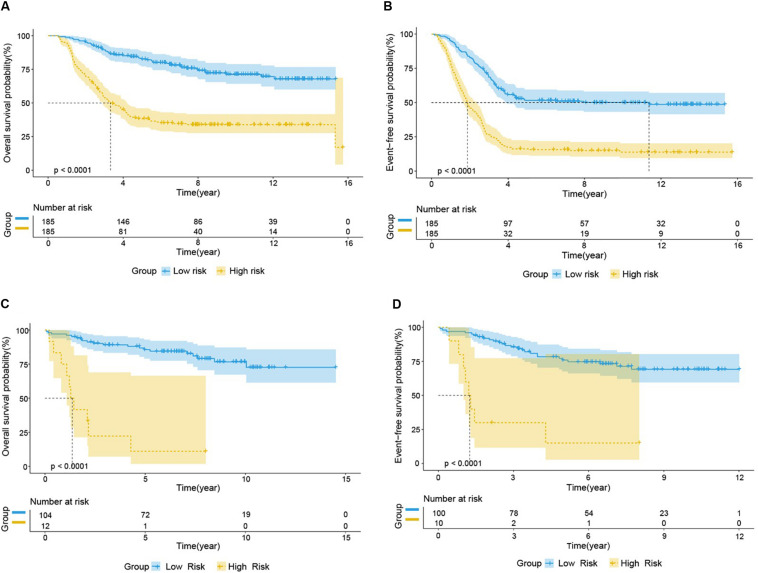
Prognostic analysis of high-risk and low-risk group in B-ALL patients. Kaplan-Meier survival curve analysis of OS in the high-risk (yellow line) and low-risk (blue line) patients in Total Set **(A)** and External Validation Set **(C)**. Kaplan-Meier survival curve analysis of EFS in the high-risk and low-risk patients in Total Set **(B)** and External Validation Set **(D)**.

We used the test set (*n* = 148), the total set (*n* = 370) and the external validation set (*n* = 116) to assess the predictive value of the risk score model constructed using the training set. We used the median value of the risk score formula defined using the training set as the threshold for distinguishing high- and low risk groups. The AUC curve showed that the 3-year, 5-year and 10-year OS of the test set, using the total set and the external validation set were 0.785, 0.764, 0.720, 0.806, 0.801, 0.761, 0.809, 0.761, and 0.739, respectively (Figures 5B–D). The distribution of risk score, survival status, and the heatmap of risk genes in internal total set and external validation set are shown in Figure 6. Among these four prognosis-related genes, *HOXA7, S100A10*, and *S100A11* were overexpressed in high risk group and *IFI44L* was downregulated. The prognosis of patients was worse with the increase in risk scores. In the total set, the mortality of patients in the high-risk group (64.3%) was higher than that in low-risk group (27.0%, *p* < 0.0001). Similar conclusions were drawn for the external validation set (mortality in high- and low-risk groups: 33.3% vs. 2.3%, *p* < 0.0001). Kaplan-Meier curves showed that patients in the high-risk group had significantly poorer OS (3-year OS for total set: 55.0% vs. 89.6%; 95% CI: 48.3–62.7%, 85.3–94.2%; 3-year OS for external validation set: 22.2% vs. 89.2%; 95% CI: 7.17–68.9%, 83.3–95.4%; 5-year OS for total set: 38.3% vs. 82.7%; 95% CI: 31.8–46.0%, 77.3–88.4%; 5-year OS for external validation set: 11.1% vs. 85.7%; 95% CI: 1.86–66.5%, 79.0–93.0%; all *p* < 0.0001) and EFS (3-year EFS for total set: 26.5% vs. 68.6%; 95% CI: 20.8–33.7%, 62.2–75.6%; 3-year EFS for external validation set: 30.0% vs. 85.5%; 95% CI: 11.6–77.3%, 78.8–92.9%;5-year EFS for total set: 15.7% vs. 51.6%; 95% CI: 11.2–21.9%, 44.8–59.5%; 5-year EFS for external validation set: 15.0% vs. 75.9%; 95% CI: 2.80–80.4%, 67.6–85.3%; all *p* < 0.0001) (Figure 7).

### The Genetic Risk Score Model Is an Independent Prognostic Factor

**FIGURE 8:**
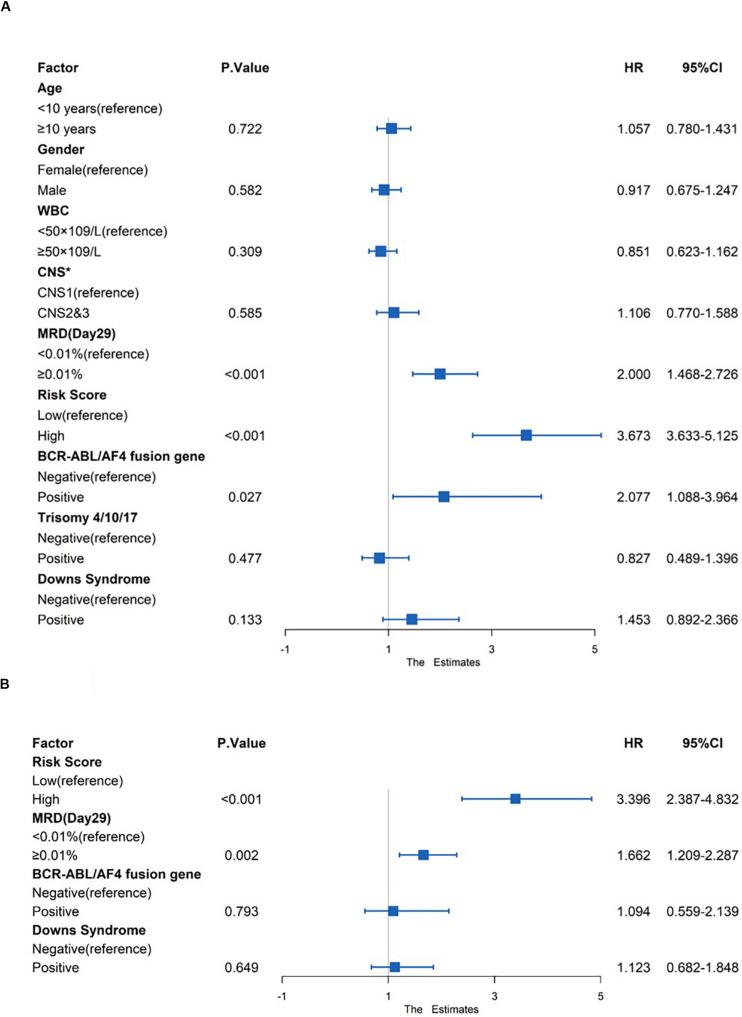
Prognostic prediction value of four-gene prognostic risk models in Total Set. **(A)** Univariate Cox regression analysis of OS. **(B)** Multivariate Cox regression analysis of OS. ‘*’ means CNS, Central Nervous System, CNS1 = CNS negative, CNS2 ≤ 5 WBC/μL in CSF with blasts, CNS3 = CNS positive.

Risk factors such as age, gender, white blood count (WBC), central nervous system (CNS) status at diagnosis, minimal residual disease (MRD) at day 29, risk score and genetic abnormalities were evaluated using univariate Cox model analysis (Figure 8A) in the total set. Only factors with a *p*-value of <0.2 (risk score, MRD at day 29, BCR-ABL/AF4 fusion gene and Downs Syndrome) in the univariate analysis were included in the multivariate analysis. Multivariate Cox regression analysis showed that high risk score and MRD ≥ 0.01% at day 29 were independent prognostic risk factors (*p* < 0.001 and =0.002; HR = 3.396 and 1.662; 95% CI were 2.387–4.832 and 1.209–2.287, respectively) (Figure 8B).

### Identification of DEMs Based on Disease Recurrence

**FIGURE 9:**
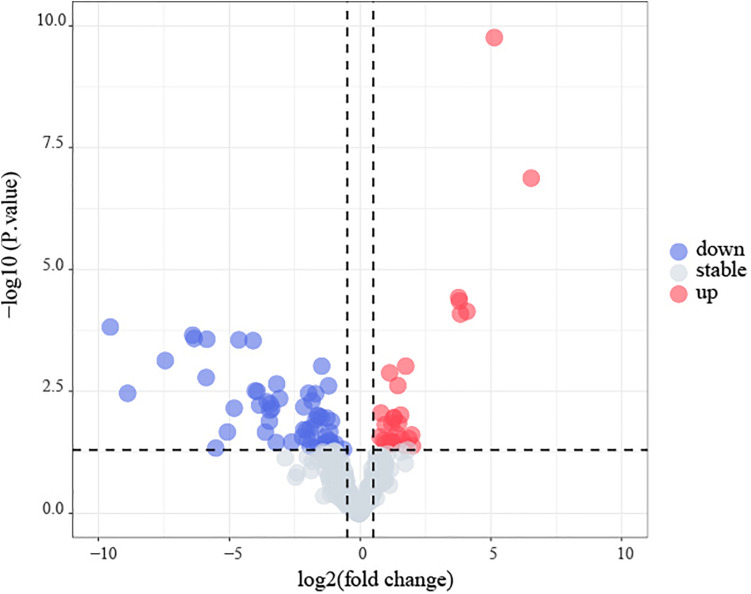
Identification of early recurrence-associated DEMs in B-cell acute lymphoblastic leukemia from TARGET database. Volcano plot of DEGs from TARGET. Genes with p < 0.05 and | log2(FC)| > 0.5 are shown in red (upregulated microRNAs) and blue (downregulated microRNAs).

Amongst the 53 B-ALL patients from the TARGET database, 17 differential microRNA precursors (pre-miRNAs) were identified based on early disease recurrence (Figure 9), including eight upregulated miRNAs and nine downregulated miRNAs (Table 5).

**TABLE 5:** The DEMs identified in 53 B-ALL patients from TARGET database.

**Gene Symbol (upregulated DEMs)**
hsa-mir-885, hsa-mir-4465, hsa-mir-3919, hsa-mir-124-3, hsa-mir-124-1, hsa-mir-124-2, hsa-mir-3662, hsa-mir-27b
**Gene Symbol (downregulated DEMs)**
hsa-mir-26a-1, hsa-mir-16-2, hsa-mir-30c-1, hsa-mir-486-2, hsa-let-7f-1, hsa-mir-103a-2, hsa-mir-1247, hsa-mir-324, hsa-mir-10b

### MiRNA Targeting DEGs

**FIGURE 10:**
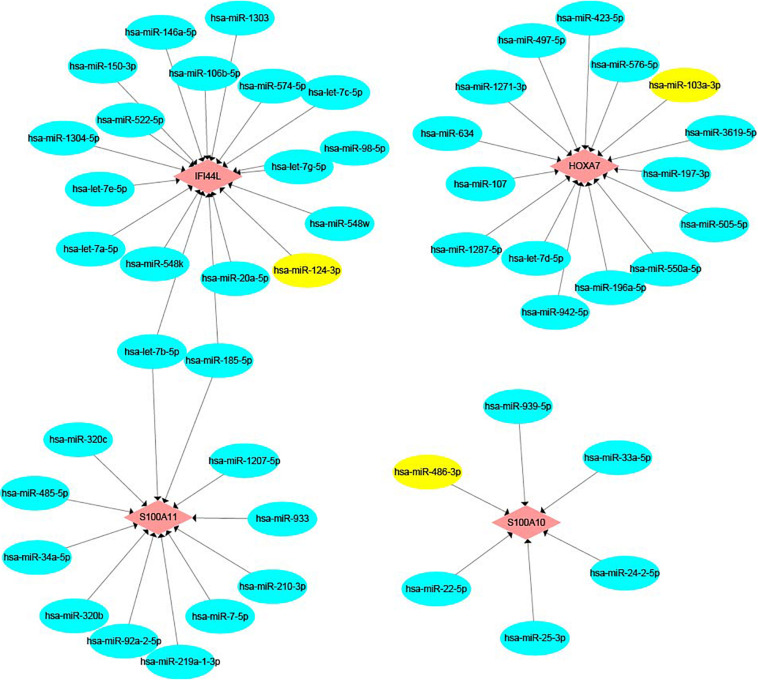
The mRNA-miRNA network. Pink color represents target genes and blue color represents miRNA. Yellow color shows hsa-miR-124-3p, hsa-miR-103a-3p, and hsa-miR-486-3p.

We built an mRNA-miRNA network using data from two prediction websites and Cytoscape. As shown in Figure 10, hsa-miR-124-3p, which is a pre-miRNA (hsa-mir-124-1, hsa-mir-124-2, and hsa-mir-124-3) were upregulated in early recurrence patients, targets the gene *IFI44L.* Hsa-miR-103a-3p and hsa-miR-486-3p have the target genes *HOXA7* and *S100A10*, respectively. These two miRNAs could be produced by shear processing of mir-103-2 and mir-486-2, both of which are downregulated in patients who relapsed within 24 months.

## Discussion

Recurrence is an important factor for the increase of mortality in ALL, and the early recurrence of leukemia predicts a worse prognosis. Therefore, the identification of factors associated with early recurrence is vital for the identification of new risk subgroups in ALL patients, and also offers new directions for targeted therapies in high-risk patients. MLL rearranged, hypodiploid, BCR/ABL fusion gene and high end-induction MRD are the common risk factors for relapse in childhood ALL patients (19). And several published articles showed that the biomarkers for early recurrence in B-ALL include the persistence of MRD, chromosome 19p13 translocations, upregulation of nucleotide excision repair genes, deletion of CDKN2A/B and overexpression of LINC00152 (Supplementary Table S1) (20–24). In this study, we looked for genes involved in early relapse of B-ALL on a larger sample. DEGs were identified from early recurrence vs. non-early recurrence groups both in the total set from the TARGET database and in the GSE13576 dataset. The common DEGs were subjected to univariate COX regression and LASSO regression in the training set. The optimal candidate DEGs were validated using the Oncomine website. We constructed a genetic risk score model of B-ALL using stepwise multivariate COX regression analysis, and verified its prognosis predictive value.

In this study, a four-gene risk score model was identified from 65 common DEGs. The AUCs of the risk model in the training and verification sets were all greater than 0.7, indicating that the risk score had a good prognosis predictive value in >70% of patients. The survival indexes OS and EFS in the high-risk group were confirmed to be worse than those in low-risk group with statistically significant differences. To further verify the relationship between the model and prognosis, we included clinical factors for univariate and multivariate COX analyses. The results showed that high genetic risk score and MRD ≥ 0.01% at day 29 were independent adverse prognostic factors.

The *HOXA7* gene is a member of the homeobox (HOX) gene family, which plays vital roles in hematopoiesis and cell differentiation (25). Previous research has shown that the gene *HOXA7* is overexpressed in MLL-fusion leukemias, which commonly have very poor outcomes (26–29). The proteins encoded by *S100A10* and *S100A11* are members of the S100 family. S100 proteins are involved in many cellular processes, such as cell growth and motility, cell cycle progression and differentiation (30). Dysregulated expression of S100 proteins is a common feature of human cancers, including pediatric ALL (31, 32). High *S100A10* expression has a link to poor outcomes and chemoresistance in several types of cancer, including leukemia (33). The overexpression of *S100A11* is also associated with cancer progression and poor survival. The *IFI44L* gene is a type I interferon-stimulated gene. Although the function of *IFI44L* is unclear, some studies have revealed that it is involved in the inflammatory response, and may be a tumor suppressor (34–36). According to data from a study by Bhojwani et al., the expression of the genes *HOXA7*, *S100A10*, and *S100A11* was significantly higher in B-ALL patients who relapsed within 24 months than in those who did not (4). In contrast, *IFI44L* was highly expressed in patients with no recurrence or late recurrence. This finding is consistent with those of our research. These results suggest the possibility of the use of the four genes as early prognostic biomarkers for childhood and adolescent B-ALL.

MiRNAs are dysregulated in cancers, including hematological malignancies (37, 38). MiR-124 is regarded as a tumor suppressor in some solid tumors (39–41). Previous studies indicated that upregulation of miR-124 can reduce the invasion ability of tumor cells (42, 43). However, the role miR-124 plays in hematological malignancies remains controversial (44). Chen et al., suggested that miR-124-1 deregulation might have a favorable impact on prognosis in AML (45). Liang found that miR-124 is overexpressed in pediatric prednisone-poor response ALL, and contributes to glucocorticoid resistance (46). MiR-103a-3p is considered to be an oncogene in gastric cancer, colorectal cancer, and breast cancer, while it suppresses cell proliferation and invasion in prostate cancer (47–49). In leukemia, the function of miR-103 is also controversial. Upregulation of miR-103 was found by Yefenof et al., to sensitize leukemia cells to glucocorticoids (50). However, a study by Zhang et al., showed that miR-103 is upregulated in adriamycin-resistant cells (51). miR-486-3p could act as a tumor-suppressive miRNA in several cancers (52–54). In our study, pre-miR-124s (miR-124-1, miR-124-2 and miR-124-3) were upregulated in early recurrence B-ALL, and miR-124-3p was predicted to target the gene *IFI44L*. pre-miR-103a (miR-103a-2) and pre-miR-486 (miR-486-2), which target the genes *HOXA7* and *S100A10*, respectively, were downregulated in patients who relapsed within 24 months. In summary, the dysregulation of microRNA may be a potential mechanism for the abnormal expression of these prognostic genes.

## Conclusion

Our study identified four genes related to early recurrence of B-ALL in childhood and adolescent patients, using integrated bioinformatics analysis. The four-gene risk score model is an independent prognostic risk indicator. Detection of the expression levels of these four genes provides a new signature for the early identification of high-risk patients.

## Data Availability Statement

The datasets presented in this study can be found in online repositories. The names of the repository/repositories and accession number(s) can be found in the article/ Supplementary Material.

## Ethics Statement

The studies involving human participants were reviewed and approved by all data are from public database on the internet. Written informed consent from the participants’ legal guardian/next of kin was not required to participate in this study in accordance with the national legislation and the institutional requirements.

## Author Contributions

YH was mainly responsible for the idea design and writing of the article. JL was in charge of the writing of the manuscript. YC was in charge of the data collection. PJ was responsible for the chart making. LW provided the statistical assistance. JH contributed to the idea design and article modification for this article. All authors contributed to the article and approved the submitted version.

## Conflict of Interest

The authors declare that the research was conducted in the absence of any commercial or financial relationships that could be construed as a potential conflict of interest.

## Footnotes

1 https://www.ncbi.nlm.nih.gov/geo/

2 https://ocg.cancer.gov/programs/target/

3 https://bioinfogp.cnb.csic.es/tools/venny/index.html

4 http://www.oncomine.org

5 http://mirwalk.umm.uni-heidelberg.de/

## Supplementary Material

TABLE S1 Risk factors of early relapse in B-ALL in published literatures.
