# Supplementary material for: Identification of Early Recurrence Factors in Childhood and Adolescent B-Cell Acute Lymphoblastic Leukemia Based on Integrated Bioinformatics Analysis
Source: Front Oncol. 2020 Sep 29;10:565455. doi: 10.3389/fonc.2020.565455 (PMC7550668; doi:10.3389/fonc.2020.565455)
Supplement: TABLE S1 — Risk factors of early relapse in B-ALL in published literatures. [file Table_1.docx]

| **Table S1. Risk factors of early relapse in B-ALL in published literatures** | | | |  |
| --- | --- | --- | --- | --- |
| **Study (year)** | **Prognostic Factor** | **Patients** | **Outcome** |  |
| Katz *et al.* (1989) | Persistence of MRD | 68 | No mention |  |
| Khalidi *et al.* (1999) | Chromosome 19p13 translocations | 7 | All patients died 7–29 months  following diagnosis |  |
| Ibrahim *et al.* (2018) | Overexpression of nucleotide  excision repair gene | 76 | Median EFS: 26.5 months |  |
|  |  |  |  |  |
| Kathiravan *et al.* (2018) | Deletion of CDKN2A/B | 75 | Median EFS: 14 months |  |
| Diego et al. (2020) | Overexpression of LINC00152 | 62 | DFS rate: 62.5% |  |
